# Supplementary material for: Precise excision of expanded GGC repeats in NOTCH2NLC via CRISPR/Cas9 for treating neuronal intranuclear inclusion disease
Source: Nat Commun. 2026 Jan 13;17:1683. doi: 10.1038/s41467-026-68385-5 (PMC12909857; doi:10.1038/s41467-026-68385-5)
Supplement: Supplementary file 1 — Supplementary Information [file 41467_2026_68385_MOESM1_ESM.pdf]

Supplementary Information for

Precise Excision of Expanded GGC Repeats in *NOTCH2NLC* via CRISPR/Cas9 for Treating Neuronal Intranuclear Inclusion Disease

Nina Xie[1,2]†, Yongcheng Pan[2]†, Huichun Tong[3], Yingqi Lin[3], Ying Jiang[4], Zhiqing Wang[1,2], Juan Wan[5], Wendiao Zhang[5], Xinhui Wang[2], Xiaobo Sun[6], Sen Yan[3], Peng Yin[3], Qiying Sun[1], Chengzhi Qi[6], Yun Tian[1], Lu Shen[2,7], Hong Jiang[2,7], Desheng Liang[4], Beisha Tang[2,5], Shihua Li[3]*, Xiao-Jiang Li[3,8]*, Qiong Liu[2]*

Correspondence to: Q. Liu (Lqiong66@csu.edu.cn);
X.J. Li (xjli33@jnu.edu.cn);
S. Li (lishihualis@jnu.edu.cn)

Supplementary Figure 1

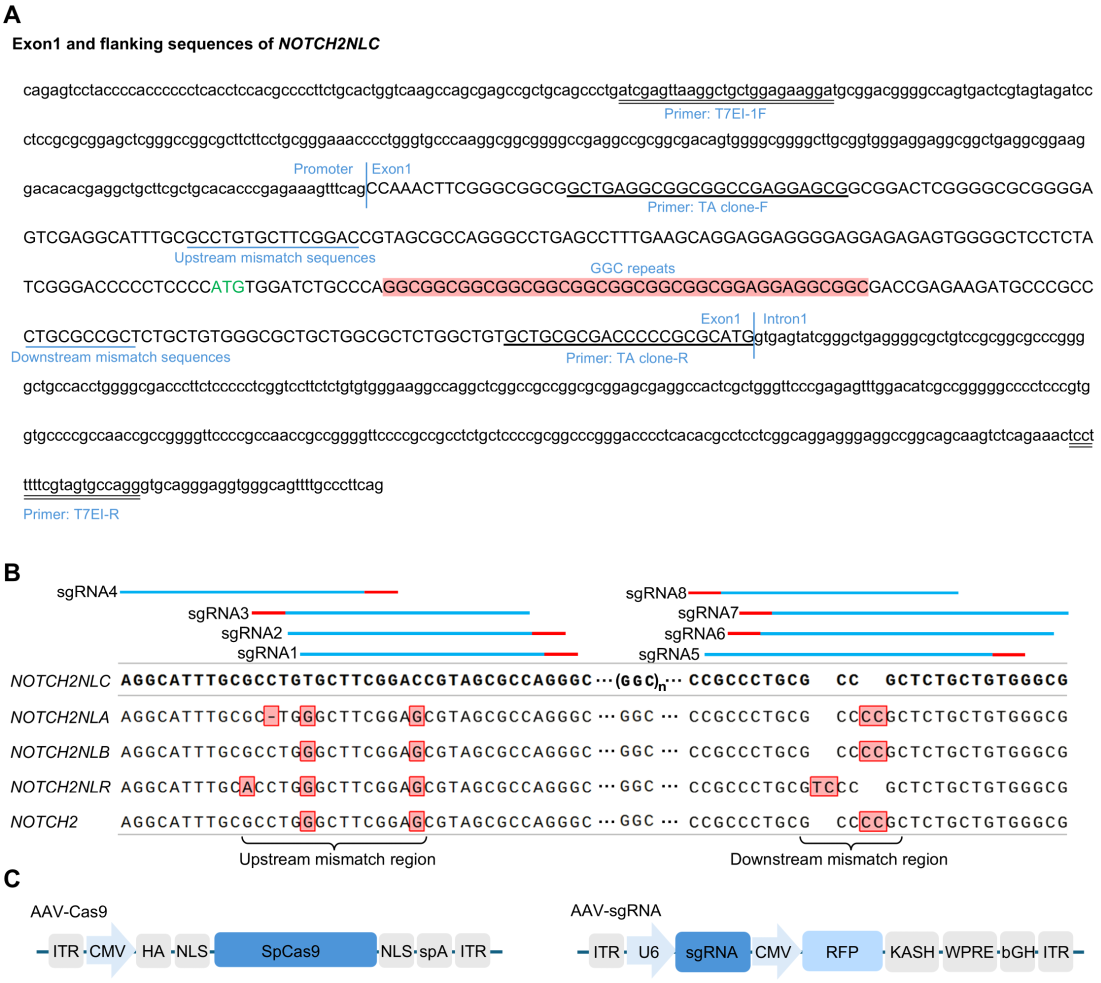

Supplementary Figure 1. Genomic DNA sequences flanking GGC repeats within *NOTCH2NLC* and sgRNA targeting sites

(**A**) Exon1 and flanking sequences of *NOTCH2NLC*. Exon 1 sequences are displayed in uppercase, with GGC repeats highlighted in red. The NLC-T7EI-F/R primer pair, which specifically amplifies *NOTCH2NLC*, was used in assessing gene-editing efficiency. The TA clone-F/R primer pair, which can amplify *NOTCH2* and *NOTCH2NL* family simultaneously, was used in assessing gene-editing specificity. (**B**) Sequence alignment of *NOTCH2* and *NOTCH2NL* family genes with sgRNA target sites. Mismatches between genes are colored red. Blue lines indicate sgRNAs; red lines indicate PAM sequences. (**C**) Schematic diagrams of Cas9 and sgRNA plasmid constructs.

Supplementary Figure 2

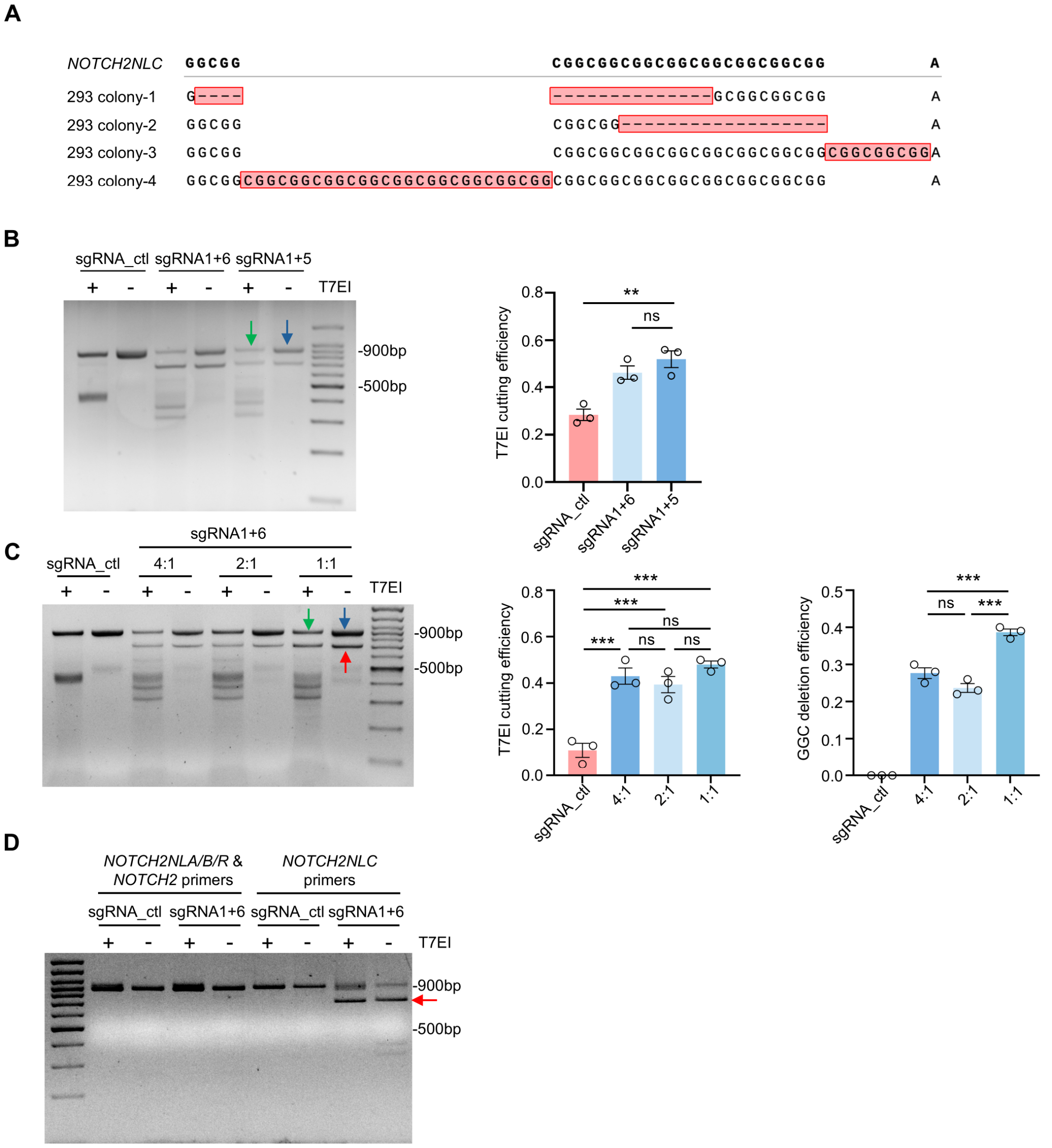

Supplementary Figure 2. Comparison of editing efficiencies of different sgRNAs at different Cas9 to sgRNA ratios in HEK293 cells

(**A**) The alignment of the sequencing results of TA cloning of different colonies revealed that the length of the GGC repeats is variable among different HEK293 cells. (**B**) T7EI assay showed that co-transfection of Cas9 and different sgRNA combinations efficiently delete GGC repeats within *NOTCH2NLC* in HEK293 cells. PCR amplification using *NOTCH2NLC* specific primers yielded a ~900 bp product, with a ~150 bp reduction observed due to a GGC repeat deletion. The T7EI cutting efficiency (%) = [1-(gray value of cut band)/ (gray value of uncut band)] *100%. The cut and

uncut bands are indicated by green arrow and blue arrow, respectively. Data are represented as mean ± SEM (N=3 independent experiments). One-way ANOVA test with multiple comparisons, $^{**}P$=0.0032, ns = not significance. (**C**) Gene-editing efficiency at different ratios of Cas9 to sgRNA. The GGC deletion efficiency (%) = [(gray value of edited band)/ (gray value of edited band + unedited band)] *100%. The edited and unedited bands were indicated by red arrow and blue arrow, respectively. Data are represented as mean ± SEM (N=3 independent experiments). One-way ANOVA test with multiple comparisons, for T7EI cutting efficiency, $^{***}P$=0.0003 (4:1 vs ctl), $^{***}P$=0.0007 (2:1 vs ctl), $^{***}P$=0.0001 (1:1 vs ctl). For GGC deletion efficiency, $^{***}P$=0.0003, $^{****}P$<0.0001. (**D**) Agarose gel electrophoresis evaluated the specificity of GGC deletion in *NOTCH2NLC*. The truncated PCR products (indicated by red arrow) were specifically detected in *NOTCH2NLC*, but were absent in *NOTCH2* and *NOTCH2NLA/B/R*. Source data are provided in this paper.

Supplementary Figure 3

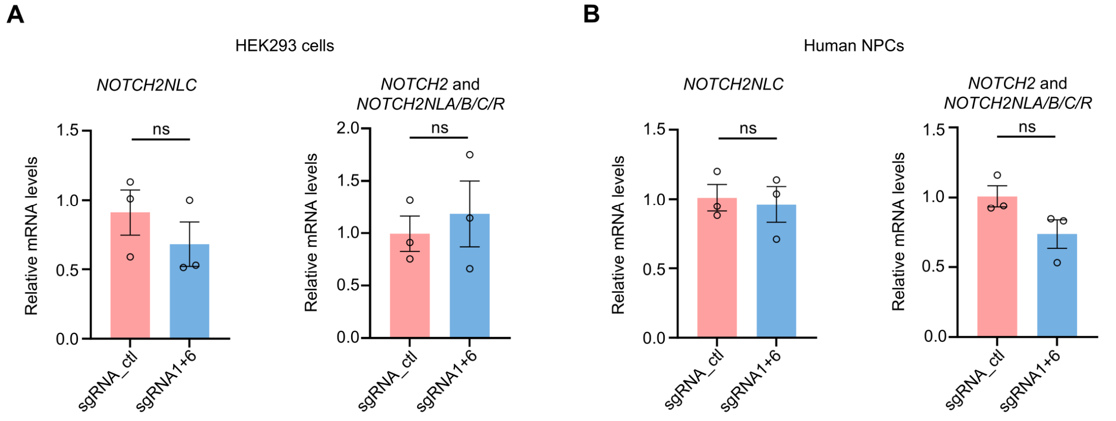

Supplementary Figure 3. Expression levels of *NOTCH2NLA/B/C/R* and *NOTCH2* after deletion of *NOTCH2NLC* GGC repeats in HEK293 cells and NPCs

(**A**) qPCR analysis on HEK293 cells that were co-transfected with Cas9 and sgRNA 1+6 or control sgRNA. (**B**) qPCR analysis on NIID NPC that were co-transfected with Cas9 and sgRNA 1+6 or control sgRNA. The expression of *NOTCH2NLA/B/C/R* and *NOTCH2* remained unchanged after deletion of *NOTCH2NLC* GGC repeats in both HEK293 cells and NPCs. Data are represented as mean ± SEM, N=3 biological replicates per group, two-tailed t-test. Source data are provided in this paper.

Supplementary Figure 4

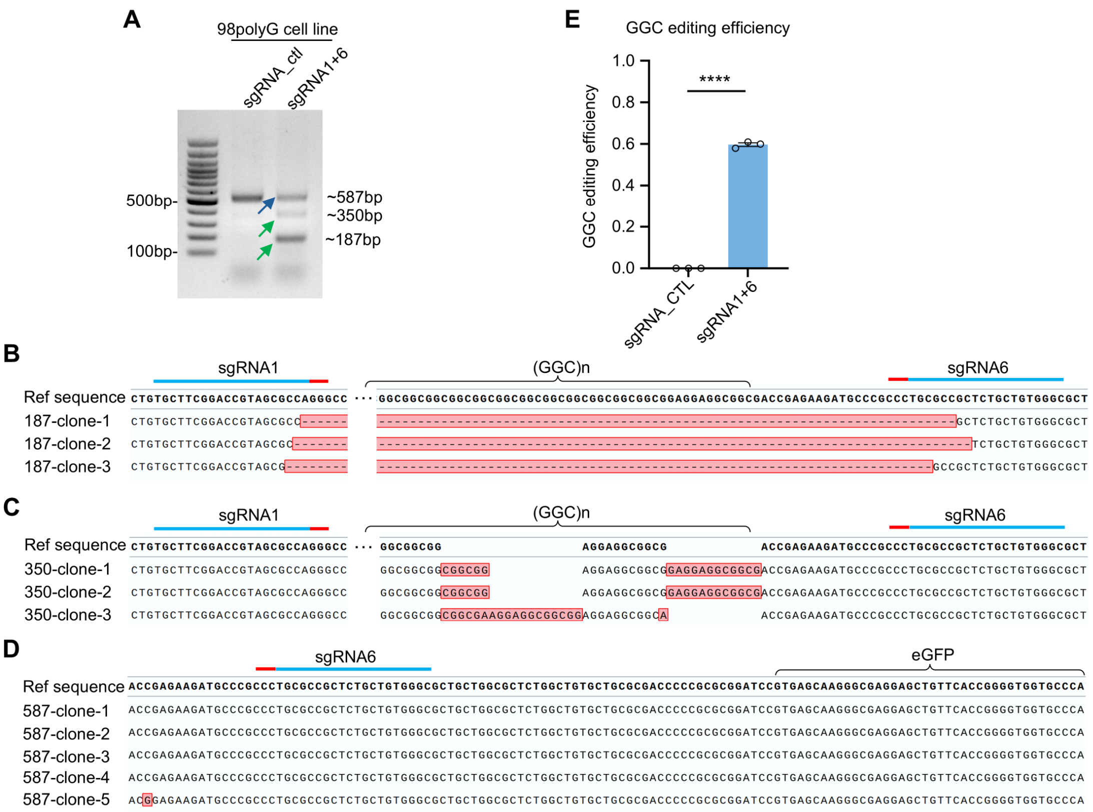

Supplementary Figure 4. Validation of Cas9-mediated GGC deletion or shortening in 98polyG cell line

(**A**) Agarose gel electrophoresis confirmed the deletion or shortening of the GGC repeat at the DNA level in the 98polyG cell line. A primer pair was designed as follows to specifically amplify the exogenous *NOTCH2NLC*-98GGC-GFP fragment without amplifying the endogenous *NOTCH2NLC* gene : the forward primer was located upstream of the upstream mismatch region, and the reverse primer was within the GFP sequence. The expected PCR ~587bp product was observed in stable 98polyG cells treated with cas9 and control sgRNA (unedited). In contrast, cells treated with cas9 and sgRNA1+6 (edited) produced three distinct bands: ~587 bp, ~350bp and ~187 bp. (**B-D**) Representative sequences from the TA cloning of the ~187 bp, ~350 bp, and ~587 bp bands were aligned with the *NOTCH2NLC*-98GGC-GFP reference sequence. The results showed that:(C) the ~187 bp product corresponded to a complete excision of the sequence between the two sgRNA cutting sites; (D) the ~350 bp product contained approximately 22 GGC repeats (within the normal range) and maintained the in-frame

GFP fusion; (E) the ~587 bp band corresponded to the unedited allele. Sequencing of the 587 bp band confirmed that none of the 15 analyzed TA clones exhibited an out-frame mutation. (**E**) The GGC editing efficiency = (gray value of two bands pointed by green arrows) / (gray value of two bands pointed by green arrows+ gray value of the band pointed by blue arrow). Data are represented as mean ± SEM, N=3 biological replicates per group, two-tailed t-test. ****P<0.0001. Source data are provided in this paper.

Supplementary Figure 5

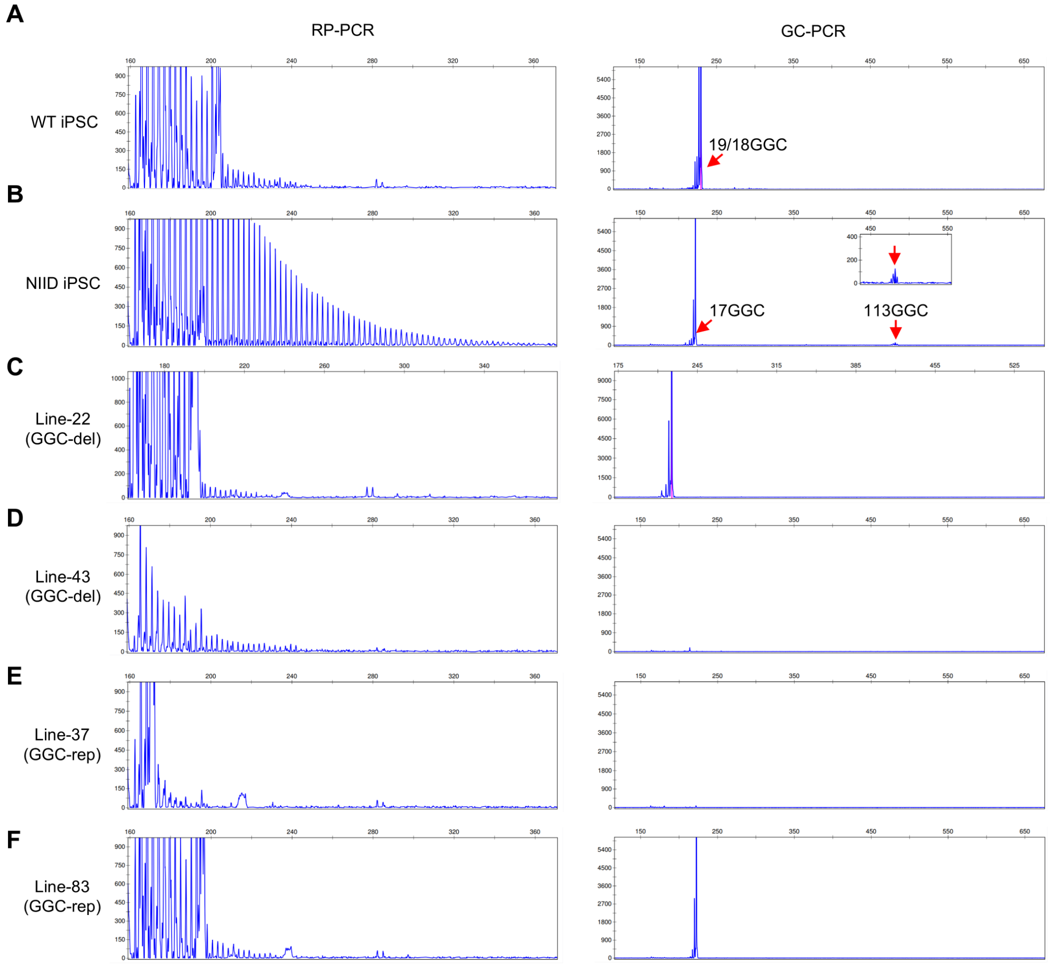

Supplementary Figure 5. Verification of GGC repeat expansions in NIID patient iPSCs pre- and post-gene-editing

(**A-F**) Representative electropherogram of repeat primed PCR (RP-PCR) and GC-rich PCR (GC-PCR) assay on WT iPSCs (A), NIID patient-derived iPSCs (B), GGC-deletion iPSCs (C-D) and GGC-repair iPSCs (E-F). Left panels: The characteristic saw-tooth tail pattern, indicative of repeat expansion in RP-PCR assay, was exclusively observed in NIID iPSCs, but absent after GGC-deletion or GGC-repair. Right panels: An abnormal peak in GC-PCR, corresponding to 113 GGC repeats, was uniquely present in NIID iPSCs but absent after GGC-deletion or GGC-repair. Inset shows magnified view of the peak.

Supplementary Figure 6

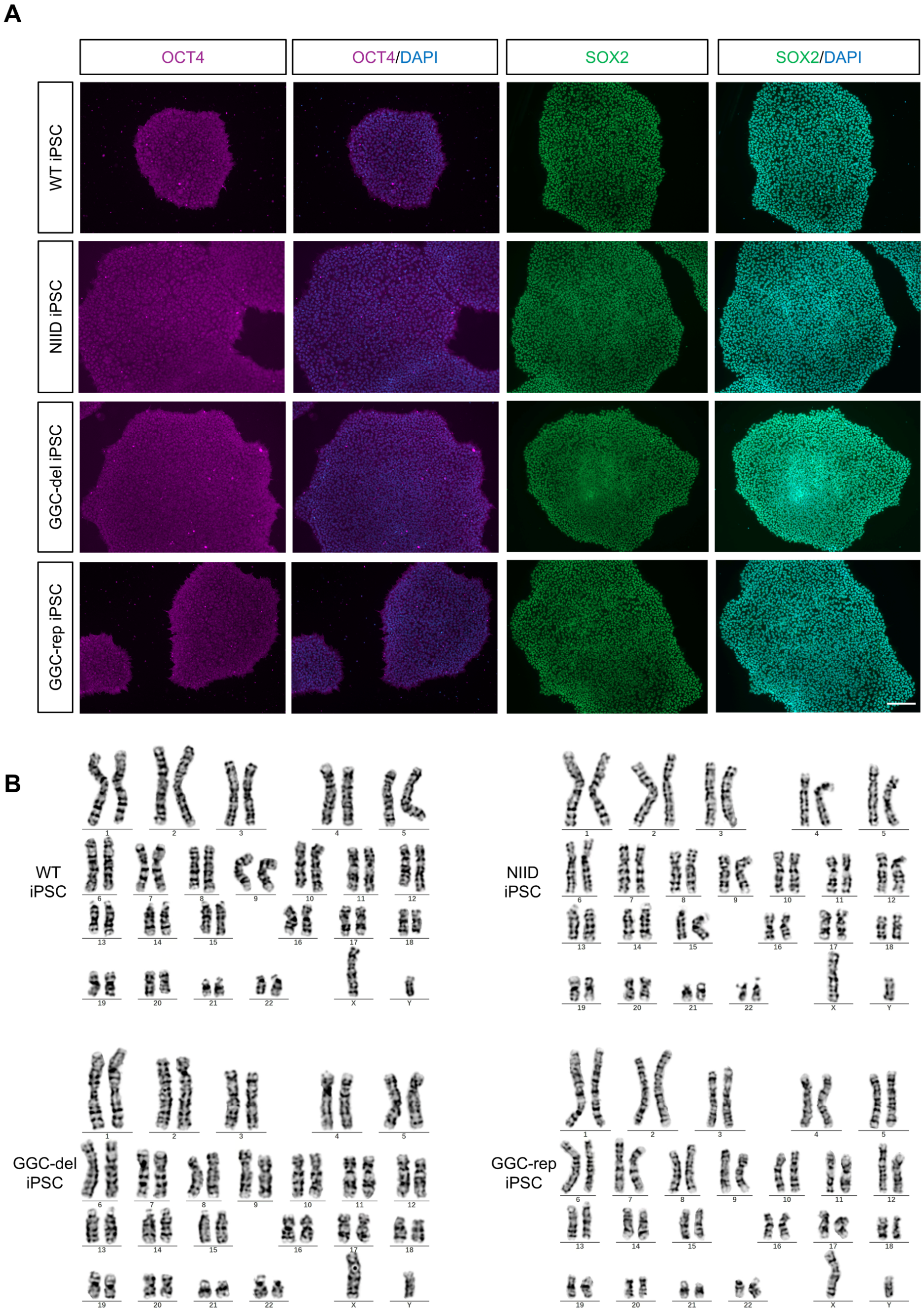

Supplementary Figure 6. Characterization of pluripotency and karyotypes of NIID iPSCs pre- and post-gene-editing

(**A**) Immunofluorescence analysis showed that GGC-deletion or GGC-repair iPSC lines retained expression of the pluripotency markers OCT4 and SOX2. (**B**) Karyotype analysis of WT iPSCs, unedited NIID iPSCs, and gene-edited NIID iPSCs (GGC-deletion and GGC-repair) showed normal Karyotype. The scale bar is 200μm.

Supplementary Figure 7

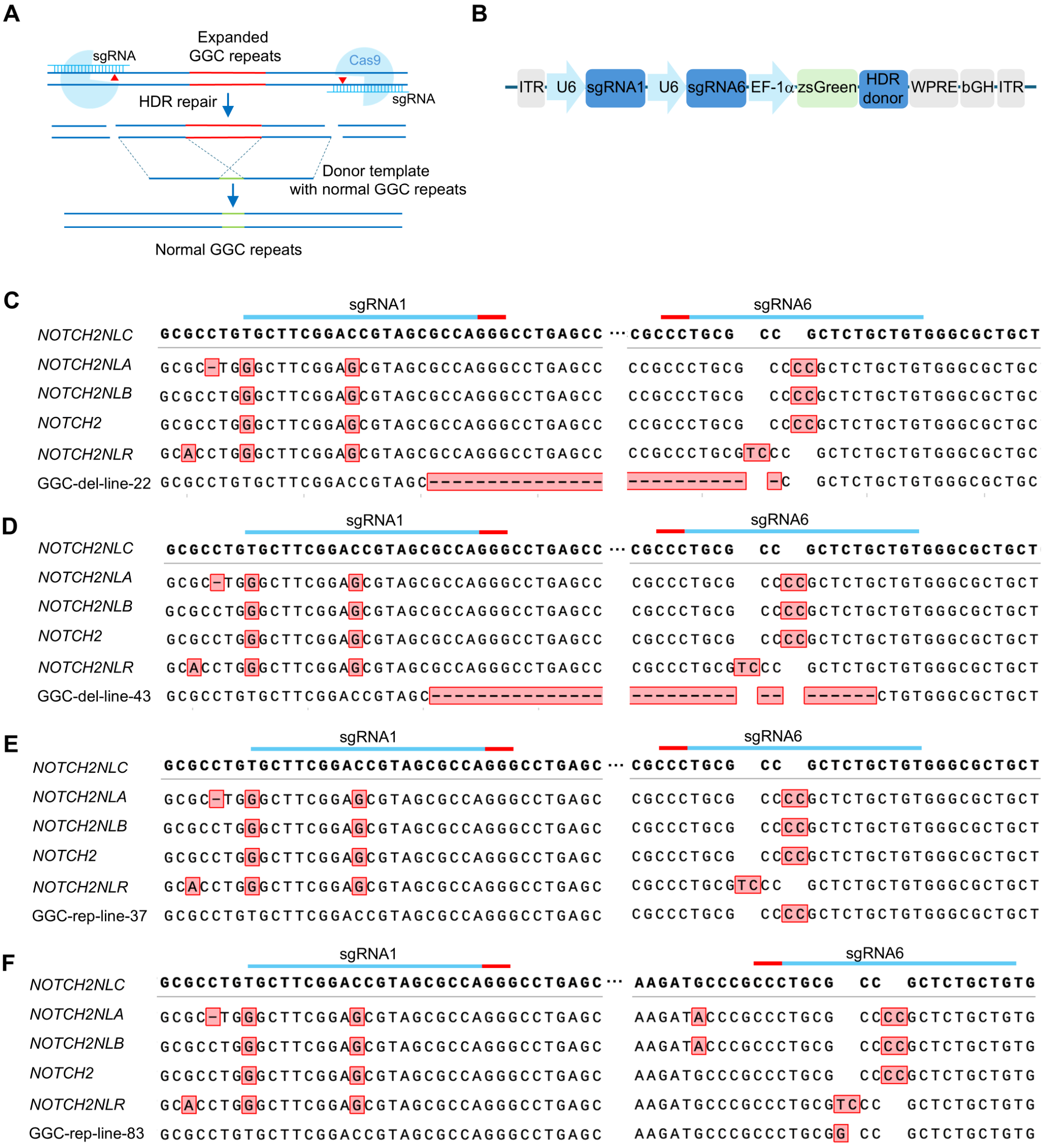

Supplementary Figure 7. Precision gene-editing strategy and sequence validation in NIID iPSCs

(**A**) Schematic of the homology-directed repair (HDR) gene-editing strategy. (**B**) Schematics of plasmid expressing sgRNAs and HDR donor for nucleofection. (**C-F**) Validation of gene-editing outcomes by TA cloning and Sanger sequencing on GGC-deletion (C-D) and GGC-repair (E-F) iPSC lines.

Supplementary Figure 8

A

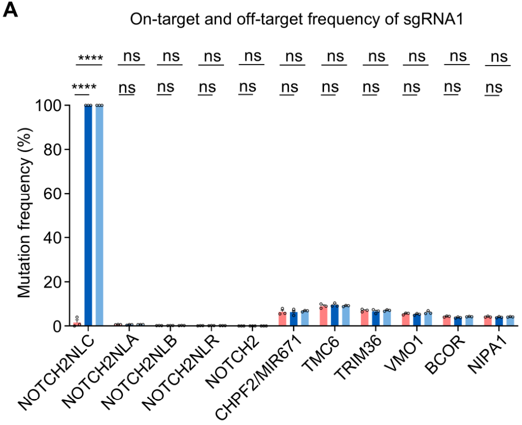

B

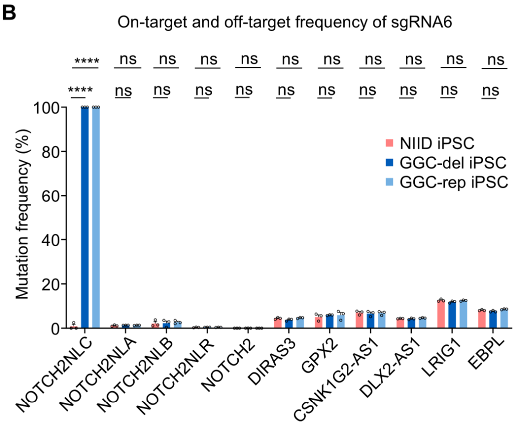

C

Variant type	Events in NIID iPSC (%)	Events in GGC-deletion iPSC (%)	Events in GGC-repair iPSC (%)
SNP	3696071±8585<br>(0.11229±0.00026082)	3705126.5±2161.5<br>(0.11256±0.000065667)	3699329±1133<br>(0.11239±0.000034421)
InDel	600816±12269<br>(0.018253±0.00037274)	617354.5±3426.5<br>(0.018756±0.00010410)	607611±6<br>(0.018460±0.00000018228)
SV	12174±817<br>(0.00036985±0.000024821)	12601±112<br>(0.00038282±0.0000034026)	11890.5±170.5<br>(0.00036124±0.0000051799)
CNV	15653±469<br>(0.00047555±0.000014248)	15572±434<br>(0.00047309±0.000013185)	15270±958<br>(0.00046391±0.000029105)
SNP in Exon	22926.5±39.5<br>(0.00069652±0.0000012)	22897.5±0.5<br>(0.00069564±0.00000001519)	22878±35<br>(0.00069504±0.0000010633)
InDel in Exon	597±3<br>(0.000018137±0.000000091142)	594.5±4.5<br>(0.000018061±0.00000013671)	592±1<br>(0.000017985±0.000000030381)
SV in Exon	606±85<br>(0.000018411±0.0000025823)	612±7<br>(0.000018593±0.00000021266)	600.5±40.5<br>(0.000018244±0.0000012304)
CNV in Exon	774.5±4.5<br>(0.00002353±0.00000013671)	789.5±9.5<br>(0.000023985±0.00000028862)	825.5±20.5<br>(0.000025079±0.0000006228)

D

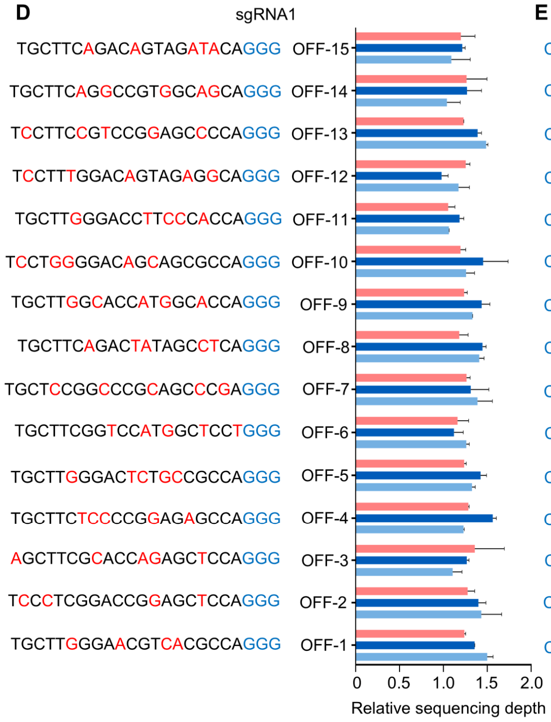

E

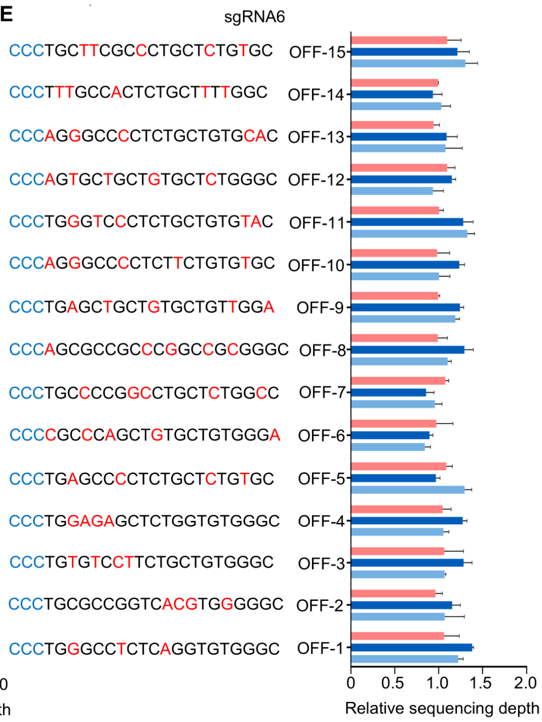

Supplementary Figure 8. Deep-sequencing and whole-genome sequencing analysis on edited and unedited iPSCs

(**A-B**) Deep-sequencing analysis revealed comparable and low off-target activity between edited (GGC-del and GGC-rep) and unedited NIID iPSC colonies for both sgRNA1 (A) and sgRNA6 (B) across the top 20 predicted off-target sites. Potential off-target sites and target loci were deep-sequenced, with mutation frequency calculated as (mutated reads/total reads) *100% at each site. Two-way ANOVA test with multiple comparisons, ****P<0.0001; N=3 per group; Data are represented as mean ± SEM. (**C**) Whole genome sequence (WGS) analysis revealed comparable and negligible extent of single-nucleotide polymorphism (SNP), insertion/deletion (InDel), structure variants (SV) and copy number variants (CNV) between edited iPSCs and unedited iPSC. The variant ratio (%) was calculated by normalizing the numbers of events to the total number of base pairs in the human genome. (**D-E**) WGS analysis showed no significant off-target activity in GGC-del and GGC-rep iPSCs. Mutations caused by Cas9 editing led to a reduced number of mapped reads, thereby a reduced relative sequencing depth. Read-depth for additional 30 predicted off-target loci by sgRNA1(D) and sgRNA6 (E) showed no significant differences between groups. Read-depth was calculated by normalizing the numbers of mapped reads in those loci to the genome-wide average of mapped reads (N=2 iPSC colonies per group). Mismatched nucleotides are indicated in red. Two-way ANOVA test with multiple comparisons. The data are presented as mean± SEM. ****P<0.0001. Source data are provided in this paper.

Supplementary Figure 9

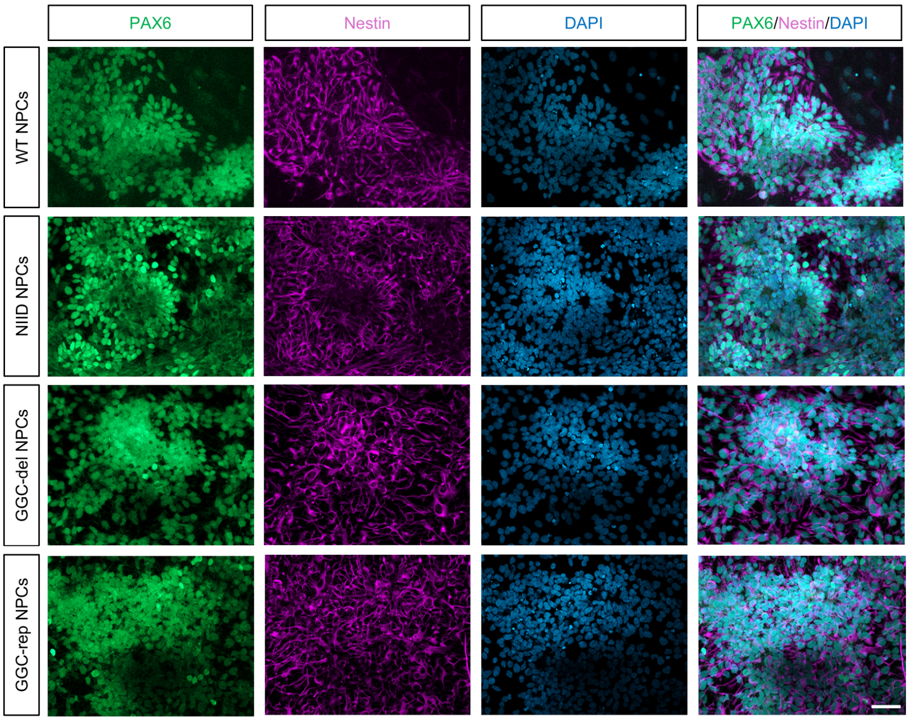

Supplementary Figure 9. Characterization of neural differentiation of NIID NPCs

Immunofluorescence showed similar expressions of PAX6 and Nestin in GGC-deletion and GGC-repair NPCs relative to WT controls. The scale bar is 50μm.

Supplementary Figure 10

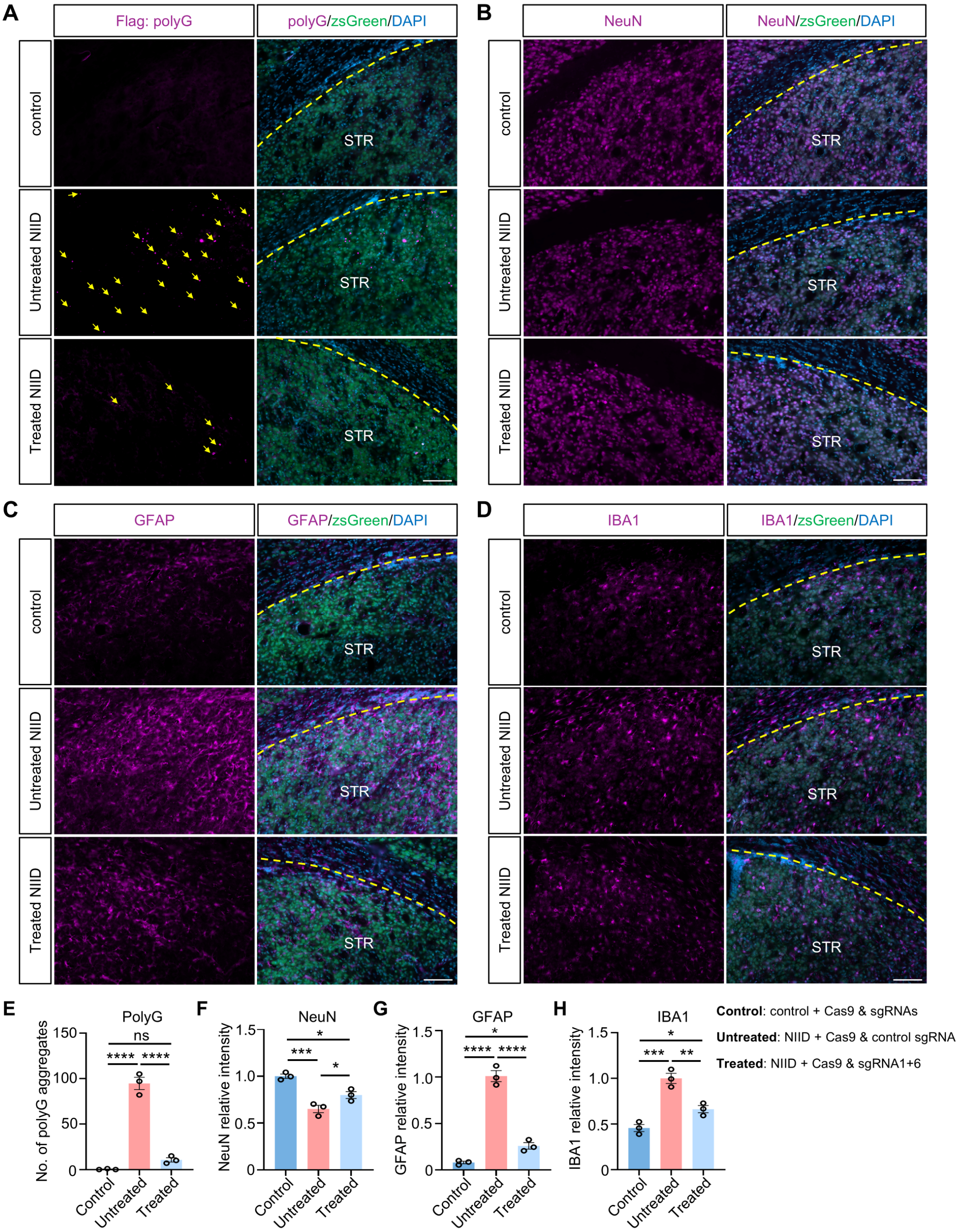

Supplementary Figure 10. Localized gene-editing of expanded GGC repeats reduced polyG level and rescued neuropathology in NIID transgenic mice

(**A**) Immunofluorescence analysis using antibody against Flag showed significant expression of polyG aggregates in the striatum of untreated NIID mice. In contrast, polyG aggregates were absent in age-matched non-disease control mice. The striatum (STR) injected with AAV-Cas9 and AAV-sgRNA1+6 (treated side) contained

significantly fewer polyG aggregates than the contralateral side injected with AAV-Cas9 and AAV-control sgRNAs (untreated side). (**B-D**) Immunofluorescence using antibodies against NeuN, GFAP, and IBA1 showed decreased NeuN (B), increased GFAP (C), and increased IBA1 (D) in the striatum of untreated NIID mice compared to age-matched non-disease control mice. Stereotaxic injection of therapeutic AAV into STR partially restored these abnormalities. The treated side showed increased NeuN (B), and decreased GFAP (C) and IBA1 (D) compared to the untreated side. ZsGreen fluorescence (reporter encoded in the AAV-sgRNA vector) confirmed efficient AAV transduction in targeted regions. Scale bar is 100 μm. ****P<0.0001; for NeuN, ***P=0.0008, *P=0.0131 (treated vs control), *P=0.0459 (treated vs untreated); for GFAP, *P=0.0482; for IBA1, *P=0.0419, **P=0.0047, ***P=0.0004. One-way ANOVA test with multiple comparisons, N = 3 mice per group. Data are represented as mean ± SEM. Source data are provided in this paper.

Supplementary Figure 11

A

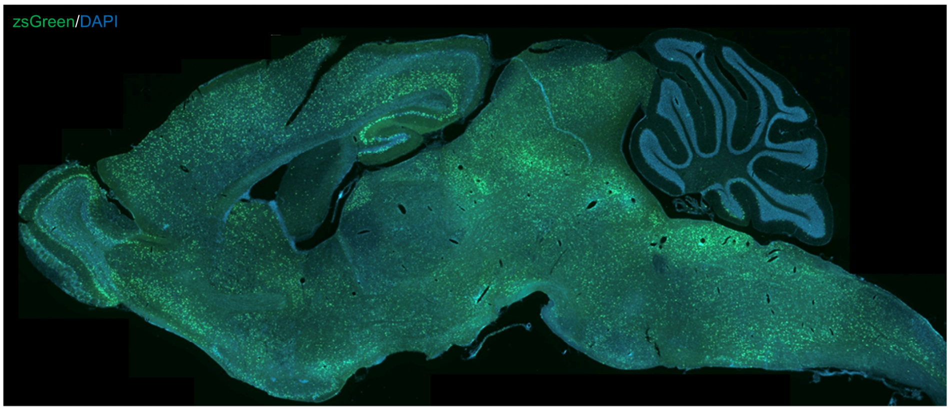

B

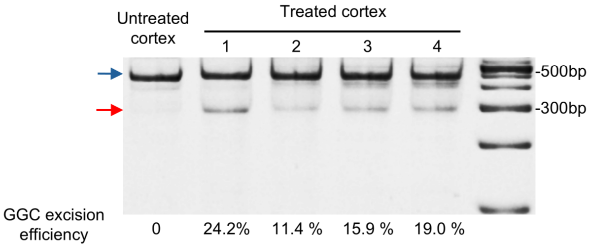

Supplementary Figure 11. Systematic gene-editing in the brain of NIID mice

(**A**) Brain distribution of zsGreen in NIID mice following retro-orbital injection of AAV-Cas9 and AAV-sgRNA. The zsGreen (reporter encoded in the AAV-sgRNA vector) confirmed efficient transduction of AAV in the whole brain. (**B**) Agarose gel electrophoresis evaluated the GGC excision efficiency in the cortex (CTX) of NIID mice following retro-orbital injection of AAV-Cas9 and AAV-sgRNA1+6 or AAV-control sgRNA. The GGC excision efficiency (%) = [gray value of edited band)/ (gray value of edited band + unedited band)] *100%. The edited and unedited bands were indicated by red arrow and blue arrow, respectively. A reduction of ~150 bp in the PCR product size was observed due to the GGC deletion.

Supplementary Figure 12

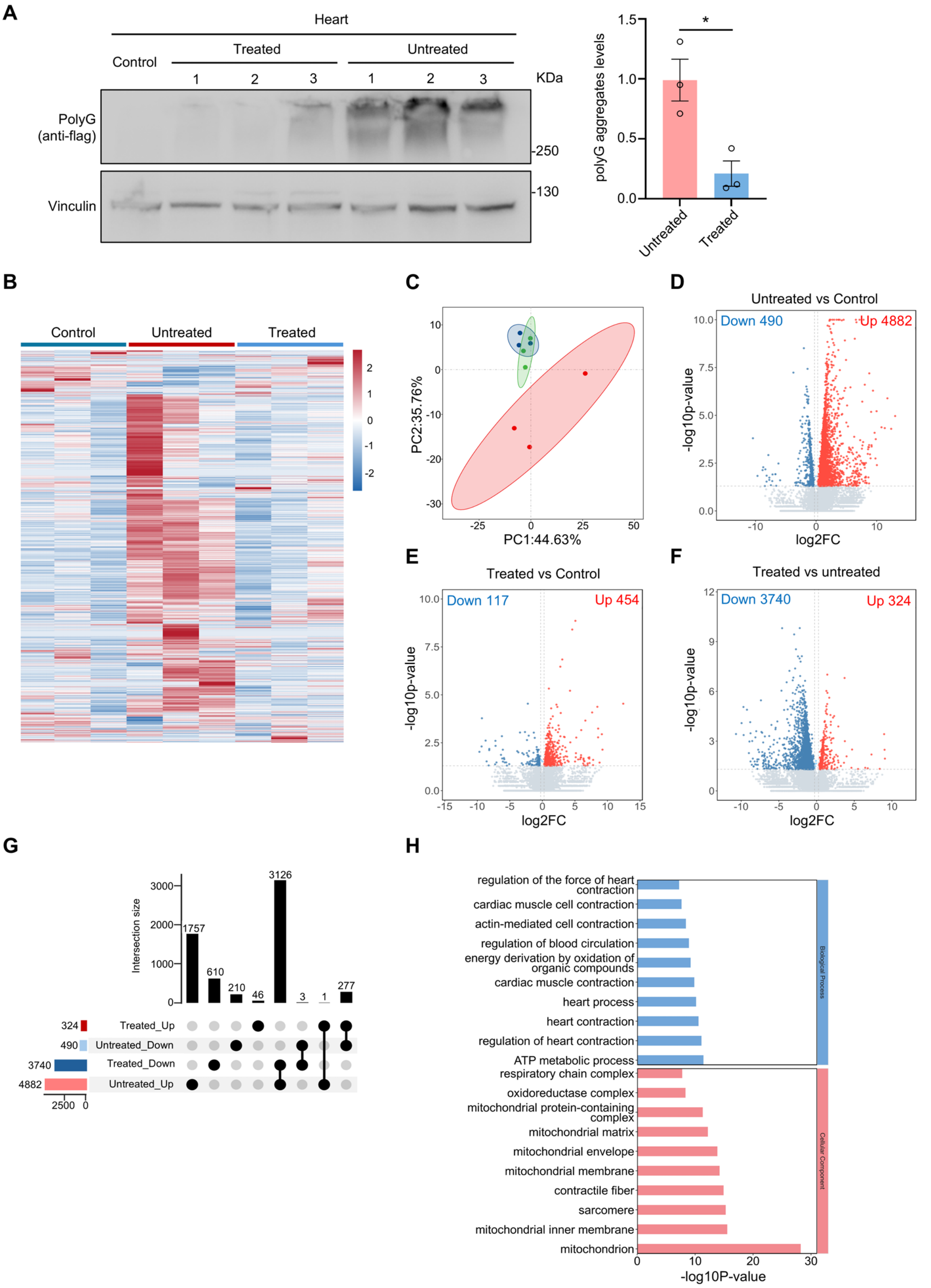

Supplementary Figure 12. Molecular analysis of heart tissues in transgenic mice after gene editing

(**A**) Western blotting of heart tissues showed that the treated NIID mice (injected with AAV-Cas9 and AAV-sgRNA1+6) via the retro-orbital injection had lower polyG levels

compared to untreated NIID mice (injected with AAV-Cas9 and AAV-control gRNAs). Littermate control mice injected with AAV-Cas9 and AAV-sgRNAs were served as negative control. Vinculin was used as the loading control. Data are represented as mean ± SEM (N=3 mice per group). Two-tailed t-test, *P=0.0186. (**B-C**) The heatmap (B) and PCA (C) analysis of RNA-seq data on heart showing transcriptomic similarity among indicated groups. The treated NIID mice exhibited gene expression patterns more similar to the control mice. (**D-F**) Volcano plots showing up-regulated (depicted in red) and down-regulated (depicted in blue) differential expressed genes among different groups. The treated NIID mice had markedly fewer DEGs than the untreated NIID mice (571 vs 5372) when compared to the control mice. (**G**) Upset plot showing the overlap and unique DEGs among different categories. 56.53% (277/490) - 64.03% (3126/4882) of dysregulated genes displayed reversal trends following treatment. The complete lists of DEGs are available in Supplementary Data 5. (**H**) GO enrichment analysis revealed that the reversed DEGs were enriched in mitochondrial function and cardiac contraction pathways. For all panels, differential expression analysis was performed using DESeq2 (two-sided Wald test), with DEGs defined as $|\log2(\text{foldchange})| \geq 0.585$ and P-value<0.05. GO enrichment was assessed using a one-sided hypergeometric test with FDR adjustment (Benjamini-Hochberg method). Source data are provided as a Source Data file.
