# Supplementary material for: Precise excision of expanded GGC repeats in NOTCH2NLC via CRISPR/Cas9 for treating neuronal intranuclear inclusion disease
Source: Nat Commun. 2026 Jan 13;17:1683. doi: 10.1038/s41467-026-68385-5 (PMC12909857; doi:10.1038/s41467-026-68385-5)
Supplement: Supplementary file 2 — Description of Additional Supplementary Files [file 41467_2026_68385_MOESM2_ESM.pdf]

Description of Additional Supplementary Files

File Name: Supplementary Data 1
Description: Information of sgRNAs designed in this study.

File Name: Supplementary Data 2
Description: Primer sequences of PCR, qPCR and Deep-seq.

File Name: Supplementary Data 3
Description: Differential expressed genes identified in NIID NPCs.

File Name: Supplementary Data 4
Description: Differential expressed genes identified in NIID mouse cortex.

File Name: Supplementary Data 5
Description: Differential expressed genes identified in NIID mouse heart.
