# Supplementary material for: Precise excision of expanded GGC repeats in NOTCH2NLC via CRISPR/Cas9 for treating neuronal intranuclear inclusion disease
Source: Nat Commun. 2026 Jan 13;17:1683. doi: 10.1038/s41467-026-68385-5 (PMC12909857; doi:10.1038/s41467-026-68385-5)
Supplement: Supplementary file 8 — Reporting Summary [file 41467_2026_68385_MOESM8_ESM.pdf]

nature portfolio

Corresponding author(s): Qiong Liu

Last updated by author(s): Dec 3, 2025

Reporting Summary

Nature Portfolio wishes to improve the reproducibility of the work that we publish. This form provides structure for consistency and transparency in reporting. For further information on Nature Portfolio policies, see our Editorial Policies and the Editorial Policy Checklist.

Statistics

For all statistical analyses, confirm that the following items are present in the figure legend, table legend, main text, or Methods section.

n/a	Confirmed	
☐	☒	The exact sample size (*n*) for each experimental group/condition, given as a discrete number and unit of measurement
☐	☒	A statement on whether measurements were taken from distinct samples or whether the same sample was measured repeatedly
☐	☒	The statistical test(s) used AND whether they are one- or two-sided<br>*Only common tests should be described solely by name; describe more complex techniques in the Methods section.*
☒	☐	A description of all covariates tested
☐	☒	A description of any assumptions or corrections, such as tests of normality and adjustment for multiple comparisons
☐	☒	A full description of the statistical parameters including central tendency (e.g. means) or other basic estimates (e.g. regression coefficient) AND variation (e.g. standard deviation) or associated estimates of uncertainty (e.g. confidence intervals)
☐	☒	For null hypothesis testing, the test statistic (e.g. F, t, r) with confidence intervals, effect sizes, degrees of freedom and P value noted<br>*Give P values as exact values whenever suitable.*
☒	☐	For Bayesian analysis, information on the choice of priors and Markov chain Monte Carlo settings
☒	☐	For hierarchical and complex designs, identification of the appropriate level for tests and full reporting of outcomes
☒	☐	Estimates of effect sizes (e.g. Cohen's *d*, Pearson's *r*), indicating how they were calculated

Our web collection on statistics for biologists contains articles on many of the points above.

Software and code

Policy information about availability of computer code

Data collection	Illumina NovaSeq X Plus / Illumina NovaSeq 6000 (sequencing platforms)<br>Agilent Bioanalyzer 2100 (library QC)<br>Zeiss Apotome 3 (imaging)<br>Bio-Rad imaging platform (chemiluminescence detection)<br>RWD stereotaxic instrument (surgery control)
Data analysis	fastp (read trimming / QC)<br>HISAT2 (RNA-seq alignment)<br>StringTie (transcript assembly / quantification)<br>DESeq2 (differential expression analysis)<br>R (statistical analysis, PCA, heatmaps, GO)<br>CRISPResso2 (editing outcome analysis)<br>CRISPOR (off-target prediction)<br>Cas-OFFinder (genome-wide off-target prediction)<br>Samtools / bcftools (WGS processing and variant calling)<br>BreakDancer, CNVnator (structural variant and CNV detection)<br>ImageJ (image quantification)<br>SnapGene (Sanger sequence alignment/ visualization)

For manuscripts utilizing custom algorithms or software that are central to the research but not yet described in published literature, software must be made available to editors and reviewers. We strongly encourage code deposition in a community repository (e.g. GitHub). See the Nature Portfolio guidelines for submitting code & software for further information.

Data

Policy information about availability of data

All manuscripts must include a data availability statement. This statement should provide the following information, where applicable:

- Accession codes, unique identifiers, or web links for publicly available datasets
- A description of any restrictions on data availability
- For clinical datasets or third party data, please ensure that the statement adheres to our policy

The raw RNA-seq data from mice have been deposited in the Gene Expression Omnibus under accession code GSE295763 [https://www.ncbi.nlm.nih.gov/geo/query/acc.cgi?acc=GSE295763]. The raw RNA-seq data from human NPCs and the raw WGS data from human iPSCs have been deposited in the Genome Sequence Archive for Human under accession code HRA011636 [https://ngdc.cncb.ac.cn/gsa-human/browse/HRA011636] and HRA014016 [https://ngdc.cncb.ac.cn/gsa-human/browse/HRA014016] respectively. The deposition and sharing of the raw data have been approved by the Human Genetics Resource Office in China (registration number: 2025BAT00839). All data needed to evaluate the conclusions in the paper are present in the paper and/or the Supplementary data files. Source data are provided with this paper.

Research involving human participants, their data, or biological material

Policy information about studies with human participants or human data. See also policy information about sex, gender (identity/presentation), and sexual orientation and race, ethnicity and racism.

Reporting on sex and gender

All participants were male. Sex determination was confirmed by karyotype analysis. No sex/gender-based analysis was performed. Written informed consent was obtained for the use of peripheral blood samples and derived iPSCs.

Reporting on race, ethnicity, or other socially relevant groupings

All participants were ethnically Han Chinese, as confirmed by government-issued identity documents.

Population characteristics

□□Control donor: 50 years old; NOTCH2NLC GGC repeats: 19/18 (within normal range).
NIID patient: 55 years old; NOTCH2NLC GGC repeats: 113/17 (pathogenic expansion in one allele).

Recruitment

Participants were recruited through outpatient clinics at Xiangya Hospital, Central South University. All individuals were sex-matched and age-matched between patient and health control to minimize confounding effects.

Ethics oversight

This study was approved by the Ethics Committee of Xiangya Hospital of the Central South University in China (Approval No. 2024040420).

Note that full information on the approval of the study protocol must also be provided in the manuscript.

Field-specific reporting

Please select the one below that is the best fit for your research. If you are not sure, read the appropriate sections before making your selection.

☒ Life sciences ☐ Behavioural & social sciences ☐ Ecological, evolutionary & environmental sciences

For a reference copy of the document with all sections, see nature.com/documents/nr-reporting-summary-flat.pdf

Life sciences study design

All studies must disclose on these points even when the disclosure is negative.

Sample size

The sample sizes used in this study were determined by established standards in the field to ensure robust and reproducible findings. For example, in behavioral test, at least eight mice per group were included ; for other experiments, at least three biological replicates were used.

Data exclusions

No data were excluded from the analyses.

Replication

For immunostaining, western blotting, and qPCR samples from at least three biological replicates were used, and the results were analyzed from at least three independent experiments.

Randomization

For in vivo experiments, allocation of littermates to treatment or control groups was random. Furthermore, the sequence of behavioral testing was randomized to prevent bias. For in vitro experiments, group allocation was determined by the experimental condition and was therefore not random. However, the order of sample processing and analysis was rigorously randomized to prevent batch effects and technical bias. For instance, in WB, while samples were loaded by group onto gels for direct comparison, the processing order of samples was randomized during preparation (lysis, sonication). Additionally, the loading order of the groups themselves was alternated across independent experimental replicates to control for any positional effects on the gel.

Blinding

To minimize bias, blinding was implemented whenever feasible. For the in vivo gene therapy and subsequent behavioral tests, investigators were blinded to both the genotype and treatment groups during the procedures and data collection. For in vitro experiments (e.g., IF, WB),

while the experimenter was aware of the groups during sample processing, the quantitative analysis of all images was performed by an investigator blinded to the sample identities.

Reporting for specific materials, systems and methods

We require information from authors about some types of materials, experimental systems and methods used in many studies. Here, indicate whether each material, system or method listed is relevant to your study. If you are not sure if a list item applies to your research, read the appropriate section before selecting a response.

Materials & experimental systems

n/a	Involved in the study	
☐	☒	Antibodies
☐	☒	Eukaryotic cell lines
☒	☐	Palaeontology and archaeology
☐	☒	Animals and other organisms
☒	☐	Clinical data
☒	☐	Dual use research of concern
☒	☐	Plants

Methods

n/a	Involved in the study	
☒	☐	ChIP-seq
☒	☐	Flow cytometry
☒	☐	MRI-based neuroimaging

Antibodies

Antibodies used

Primary antibodies used in this study include the following: Flag (Sigma-Aldrich, F1804, 1:1000), vinculin (Sigma-Aldrich, V9131, 1:1000), NeuN (Cell Signaling Technology, 24307s, 1:1000), GFAP (Cell Signaling Technology, 3670S, 1:1000), IBA1 (Wako, 019-19741, 1:1000), GFP (Invitrogen, A-11122, 1:2000), zsGreen (Sangon, D199984, 1:2000), OCT4 (Proteintech, 60242-1-Ig, 1:200), SOX2 (Proteintech, 11064-1-AP, 1:1000), PAX6 (Abcam, AB195045, 1:350), Nestin (CST, 33475S, 1:2000). Fluorescent secondary antibodies (1:200) used were donkey anti-rabbit and donkey anti-mouse antibodies conjugated with Alexa Fluor 488 or 594 from Jackson ImmunoResearch. HRP-conjugated secondary antibodies (1:10000) were donkey anti-rabbit and donkey anti-mouse from Jackson ImmunoResearch.

Validation

All antibodies used in this study have been validated by manufacturers or validated in published scientific literature.

Eukaryotic cell lines

Policy information about cell lines and Sex and Gender in Research

Cell line source(s)

HEK293: purchased from a commercial supplier (originally sourced from ATCC, Procell, CL-0001).
iPSC lines: established from peripheral blood mononuclear cells (PBMCs) of normal control and NIID patient (both are male).

Authentication

The HEK293T cell line was authenticated by short tandem repeat (STR) profiling.
Karyotyping confirmed a normal chromosomal constitution in the iPSC lines, and GGC repeat were confirmed by RP-PCR and GC-PCR.

Mycoplasma contamination

All cell lines were tested negative for mycoplasma contamination.

Commonly misidentified lines
(See ICLAC register)

No commonly misidentified cell lines were used.

Animals and other research organisms

Policy information about studies involving animals; ARRIVE guidelines recommended for reporting animal research, and Sex and Gender in Research

Laboratory animals

The conditional transgenic NOTCH2NLC-98GGC mice, generated in C57BL/6J mouse background, were crossed with EIIa-Cre mice to NOTCH2NLC-98GGC ubiquitously. The EIIa-Cre mice were obtained from the Jackson Laboratory and backcrossed into the C57BL/6 background over thirteen generations. The detailed age of mice were described in methods or figure legend.

Wild animals

This study did not involve wild animals

Reporting on sex

Sex consideration: No sex-based differences were observed in mouse behavioral or pathological phenotypes.
Experimental design: All experiments were conducted without sex stratification.
Justification: Since preliminary data showed no significant sex effects, analyses were not separated by sex.

Field-collected samples

All mice were bred and maintained on a 12:12 h light/dark cycle in the animal facility under specific pathogen-free conditions.

Ethics oversight

All animal experiments were performed in compliance with the Animal Ethics Guidelines of Xiangya Hospital, Central South University (Approval No. 202103138)

Note that full information on the approval of the study protocol must also be provided in the manuscript.

Plants

Seed stocks	Not applicable
Novel plant genotypes	Not applicable
Authentication	Not applicable
